# Vertical Root Fracture in Non-Endodontically and Endodontically Treated Teeth: Current Understanding and Future Challenge

**DOI:** 10.3390/jpm11121375

**Published:** 2021-12-16

**Authors:** Wan-Chuen Liao, Chi-Hung Chen, Yu-Hwa Pan, Mei-Chi Chang, Jiiang-Huei Jeng

**Affiliations:** 1 School of Dentistry, College of Medicine, National Taiwan University, Taipei 100229, Taiwan; wanchuen0219@hotmail.com (W.-C.L.); rudychen66@gmail.com (C.-H.C.); 2 Department of Dentistry, National Taiwan University Hospital, Taipei 100229, Taiwan; 3 Department of Dentistry, Chang Gung Memorial Hospital, Taipei 105406, Taiwan; shalom.dc@msa.hinet.net; 4 School of Nursing, Chang Gung University of Science and Technology, Taoyuan 333324, Taiwan; 5 School of Dentistry, College of Dental Medicine, Kaohsiung Medical University, Kaohsiung 807378, Taiwan; 6 Department of Dentistry, Kaohsiung Medical University Hospital, Kaohsiung 807377, Taiwan

**Keywords:** vertical root fracture, diagnosis, endodontically treated teeth, clinical features, treatment, vital root fracture

## Abstract

A vertical root fracture (VRF) is a complex complication that usually leads to tooth extraction. The aim of this article is to review the prevalence, demography, distribution, diagnostic methods, etiology and predisposing factors, clinical features, radiographic characteristics and treatment strategies of VRFs in non-endodontically treated teeth (VRFNETT) and endodontically treated teeth (VRFETT). Search terms for each subject related to VRFNETT and VRFETT were entered into MEDLINE, PubMed and Google Scholar. Systematic reviews, retrospective cohort studies, demographic research, clinical studies, case reports and case series were reviewed. Most of the VRFs were found in patients older than 40 years old. Older populations were discovered in the non-endodontically treated VRF group when compared to the endodontically treated VRF group. Male patients were found at a greater prevalence than females in the non-endodontically treated VRF group. The initial occurrence of a VRF may accompany radiolucent lines within the root canal, unusual space between the canal wall and intracanal material, a widening of the PDL space along the periradicular surfaces, angular bony destruction, step-like bone defects, V-shaped diffuse bone defects, or root resorptions corresponding to the fracture line before the clear separation of the fractured fragment. The indicative clinical and radiographic signs of VRF included a coronally positioned sinus tract, deep-narrow periodontal defects, the displacement of a fractured fragment, periradicular radiolucent halos and the widening of the root canal space. Interestingly, VRFNETT are more often observed in the Chinese population. Some patients with multiple VRFs were observed, suggesting possible predisposing factors in genetics and tooth development. The management of a VRF usually involves a multidisciplinary approach. The common distribution and features of VRFNETT and VRFETT were elucidated to facilitate recognition and diagnosis. Besides extraction, variable therapeutic schemes, such as the repair of the VRF, root amputation and others reported in earlier literature, are available. A long-term prognosis study of the various therapeutic strategies is needed.

## 1. Introduction

A vertical root fracture (VRF) is defined as a longitudinally oriented fracture of the root [1]. Clinical detection of this phenomenon is challenging, not only for general practitioners, but also for endodontic specialists. A VRF usually occurs in endodontically treated teeth, but it has occasionally also been reported in non-endodontically treated teeth [2,3]. VRFs in non-endodontically treated teeth (VRFNETT) may be an underdiagnosed entity and deserve more of our attention. Its symptoms and signs may mimic endodontic or periodontal diseases [4]. A definitive diagnosis is often difficult and accompanied with some uncertainty. Based on the improvement of diagnostic tools and dental materials, treatment alternatives to extraction have been explored.

VRFs are one of the most difficult clinical problems to diagnose and manage. The identification and treatment of a VRF requires more interpretations. VRFs in endodontically and non-endodontically treated teeth has scarcely been investigated and compared at the same time. The aim of this article is to construct a narrative review of the prevalence, demography, distribution, diagnostic methods, etiology and predisposing factors, clinical features, radiographic characteristics and treatment options of VRFs in endodontically and non-endodontically treated teeth. In order to improve our understanding of VRFs, we compared the different characteristics of VRFs in endodontically and non-endodontically treated teeth.

## 2. Methods

An electronic search was undertaken for English language articles published from 1978 until 2021. The search terms for each aspect of VRFNETT and VRFs in endodontically treated teeth (VRFETT) were entered into the following databases: MEDLINE, PubMed and Google Scholar. The inclusion criteria were systematic reviews, retrospective cohort studies, demographic research, clinical studies, case reports and case series written in English. The exclusion criteria were in vitro, ex vivo and animal model studies. The literature retrieved was screened independently by two researchers. All titles, abstracts and full texts were reviewed for the inclusion and exclusion criteria. Disagreements regarding the inclusion or exclusion of the retrieved studies were resolved following discussion between the 2 researchers. Total 106 articles were included for this narrative review. The data collection from patients with VRFETT and VRFNETT at the Dental Department of the National Taiwan University Hospital was approved by the Ethics Committee of the National Taiwan University Hospital, Taipei, Taiwan. Some relevant cases are shown in the figures of this article. This article provides a valuable clinical overview for practitioners on the basis of current knowledge and therapeutic schemes, with plenty of information as a reference guide, in managing a VRF.

## 3. Prevalence

More VRFs were identified in endodontically treated teeth [2,5,6]. The prevalence of VRFs is about 3.69–25% in endodontically treated teeth [7,8,9,10,11,12]. However, several studies have discovered the relatively lower prevalence of 2–5% [8,13,14]. This may be explained by the difficulties in the actual diagnosis of a VRF. Some patients whose endodontic treatment failed probably did not return for recall [7,10,15,16,17]. As a result, the ambiguous clinical and radiographic presentations and different inclusion criteria of the studies have led to the variable prevalence of VRFs [1].

VRFNETT have mainly been reported in Chinese patients; 40% of the fractures were discovered in non-endodontically treated teeth during a survey of 315 VRFs in Chinese patients [4]. Another study identified that 80% of 51 VRFs cases were non-endodontically treated in Chinese patients [3]. VRFNETT have seldom been discovered or published on in Western residents. The actual reasons for the differences between Eastern and Western populations are not clear. The roles of racial predilection and genetic factors await further investigation.

## 4. Demography

### 4.1. Gender

Considerable numbers of female and male patients have been reported with VRFETT. Some studies have reported more female patients [1,6,18], while others more male than female [4]. There might be no gender preference in VRFETT.

Males showing VRFNETT were found to be more common than female patients [2,19,20]. This may be because males often exhibit stronger masticatory forces and chew harder food than females [4], thus leading to a higher possibility of VRFNETT.

### 4.2. Age

Most VRFs occurred in patients aged between 30 and 69 years old [3,4,14,19]. A demographic analysis illustrated that 86.79% of the patients were older than 40, which was a significant factor [6]. Older patients have more chances of receiving extensive restorations leading to a weakening of the tooth structure [21].

VRFNETT usually occur in older populations when compared to the endodontically treated group [2,3,19,20]. The average ages were 69 years old in the non-endodontically treated group and 56 in the endodontically treated group [22], suggesting that endodontic treatment is a predisposing factor for a VRF. The teeth of the older people may sustain higher occlusal forces and more prolonged stress over time, which may lead to VRFs even without endodontic treatment [2,22]. Another possible explanation is that endodontic treatment procedures weaken the tooth structure and cause a VRF even in younger patients [4,22].

## 5. Tooth and Root Distribution of VRF

Based on demographic research, maxillary premolars and mandibular molars were found to be the most frequently fractured teeth in cases of VRFETT [6,23,24,25].

VRFNETT were often found in maxillary and mandibular first molars in the Chinese population [2,3,4,19,20,26]. Severely attrited first molars without or with minimal restorations were a common feature [2,20].

**Figure 1:**
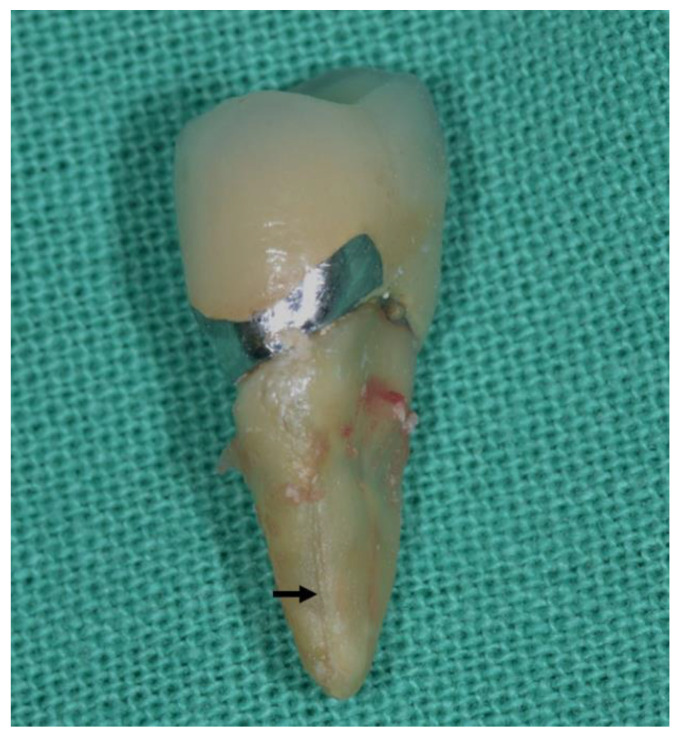
Roots with a cross-section of a smaller mesiodistal diameter and a deep oval or flattened shape are more susceptible to VRFs. The black arrow indicated the appearance of VRF.

In both VRFETT and VRFNETT, roots with a cross-section of a smaller mesiodistal diameter and with a deep oval or flattened shape (Figure 1) are more susceptible to VRFs [4,27]. VRFs were, thus, mainly detected in the maxillary premolars and mesial roots of mandibular molars [28].

## 6. Diagnostic Methods

Diagnosis should combine the patient’s subjective complaints and objective evaluations, rather than a single pathognomonic result [6]. The early diagnosis of a VRF is important to avoid unnecessary nonsurgical retreatment, continued soft tissue swelling, bone loss, or apical surgery [29], leading to difficulty in subsequent implant surgery. The possible diagnostic signs and methods are listed for clinical verification.

### 6.1. Coronally Positioned Sinus Tract

When a sinus tract is found in a VRF tooth, it is usually located in the coronal rather than apical area [28]. This type of sinus tract was found in 35–42% of VRFs [14,30]. Multiple sinus tracts are also a common feature [31,32].

### 6.2. Biting Pain and Bite Test

In order to reproduce the discomfort of the patient while chewing and thus reconfirm their chief complaint, a bite test was suggested [33]. Tools such as rubber wheels or a Tooth Slooth^®^ Fracture Detector (Professional Results Inc., Laguna Niguel, CA, USA) can be applied [33]. Endodontically treated teeth with a good quality root canal filling that exhibited specific biting pain is regarded as highly suspicious [34].

### 6.3. Deep Periodontal Probing Depth

**Figure 2:**
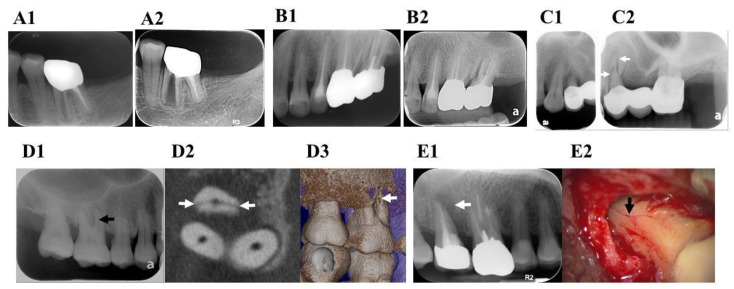
Diagnostic methods of vertical root fracture (VRF). (**A1**) 75-year-old female with VRF of the 36th mesial root in July 2018. The radiograph showed root displacement, but no periodontal pocket or soft tissue swelling. (**A2**) Swelling in the lingual side with deep pocket was noted over the 36th on the lingual side in April 2021; (**B1**) VRF of the 27th mesiobuccal root was noted on the periapical radiograph, but no swelling, periodontal pocket, or other symptoms were present in 2019. (**B2**) No symptom, soft tissue swelling, or deep pocket were noted even after follow-up for 2 years in March 2021. (**C1**) The maxillary left first premolar did not show obvious VRF in this radiograph. (**C2**) From another angle, the fracture lines were evident (white arrows). (**D1**) Radiographic image of the maxillary right first molar. There was suspicious widening of the root canal at the mesiobuccal root (black arrow). (**D2**) After performing CBCT, a fracture line was observed at the mesiobuccal root (white arrows). (**D3**) The recombination image also showed a VRF in the mesiobuccal root (white arrow). (**E1**) Radiographic image of maxillary right second molar with periradicular radiolucency (white arrow). (**E2**) After surgical intervention, VRF was observed on the root surface under microscope (black arrow).

A deep periodontal pocket is a common sign, reported in 64–93% of VRFs [22,35]. Unlike periodontal diseases, here, a deep probing depth has been found corresponding to the root fracture line. A deep narrow periodontal defect suggests underlying bony destruction caused by a VRF [28,33]. In the early stages of a VRF, some cases did not show deep probing, but the fractured line could be detected using periapical radiographs (Figure 2A1,A2,B1,B2).

### 6.4. Pulp Vitality Test

VRFNETT may show vital or necrotic pulpal responses [22]. The nerve tissue may necrotize when the fracture lines progressively extend into the pulp. A VRF is highly suspected in nonvital teeth with an intact structure or minimal restorations, when no other evident etiology can be identified [33].

### 6.5. Magnification

A microscope may assist in identifying the fracture line during nonsurgical or surgical endodontic/periodontal treatments. Magnification and direct light sources are helpful [36].

### 6.6. Radiographic Assessment

Although radiographic images do not always reveal a clear vertical fracture line, X-rays are still necessary. Employing different X-ray angles may reveal the fracture line (Figure 2C1,C2). If the fractured root fragment is displaced from the original tooth structure, then a definite diagnosis of root fracture can be made [36].

Superimposition and distortion are the most common problems encountered with two-dimensional radiographs. Cone beam-computed tomography (CBCT) images could assist in the verification of VRFs [37,38,39]. However, radiopaque intracanal materials may result in artifacts or obscure the fracture line, thus limiting its diagnostic value [36,40]. An in vivo study analyzed the accuracy of high-resolution CBCT used for detecting VRFs and concluded that the tool was non-diagnostic. Intracanal metal posts and multirooted teeth limited the diagnostic outcome [40]. Thus, CBCT is more useful in the diagnosis of non-endodontically treated VRFs [41] (Figure 2D1–D3), otherwise materials must be removed before performing the CBCT. There is still no consensus on the accuracy of CBCT in detecting endodontically treated VRFs. The voxel size also plays an important role in the observation of fracture lines. In a study assessing VRFETT via micro-computed tomography, a 9-micrometer voxel size was recommended for accurately observing a VRF [29]. The smallest currently used voxel size for CBCT is not comparable to that used for micro-computed tomography [40]. Thus, limitations remain when detecting VRFs via CBCT.

### 6.7. Exploratory Surgery

**Figure 3:**
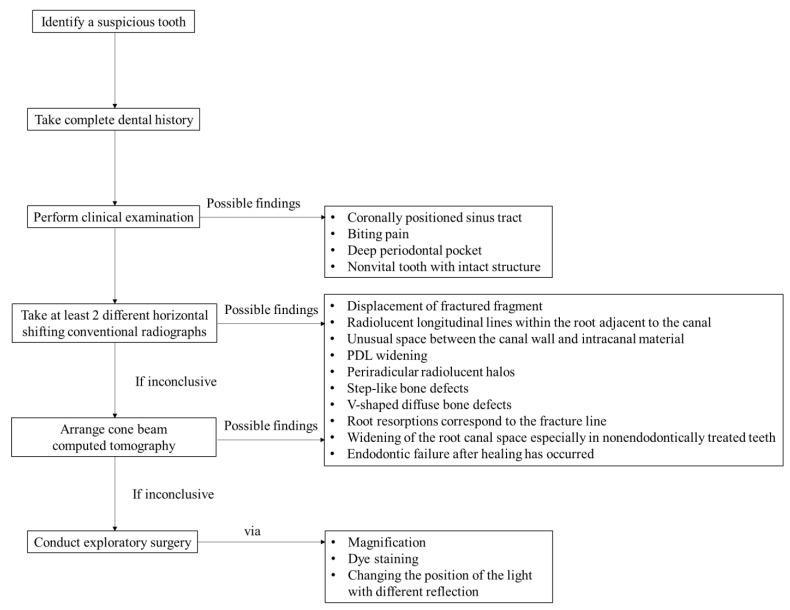
Diagnostic flowchart for the detection of VRF.

Surgical intervention is suggested when a VRF is highly suspected but cannot be confirmed through other examinations [36]. During surgery, a sharp explorer or methylene blue staining may be used to detect a possible VRF. Changing the position of the light and employing different reflections is sometimes useful when trying to observe the fracture line [32]. Many studies have concluded that direct visualization of the VRF via exploratory surgery is the gold standard [4,13,31,33,36,42]. If the clinical and radiographic examination results are inconclusive, exploratory surgery is an option (Figure 2E1,E2). The diagnostic procedures are illustrated as a flowchart in Figure 3.

## 7. Etiology and Predisposing Factors

### 7.1. Iatrogenic Factors

#### 7.1.1. Excessive Tooth Structure Removal or Over-Preparation during Root Canal Instrumentation

Excessive tooth structure removal could result in the weakening of the tooth and increase the occurrence of VRFs [28,33]. Dentinal defects, such as craze lines or incomplete fractures, may be generated during these procedures [43,44]. These cracks may initiate and lead to further root fractures. The root thickness following dentin removal is intrinsic to withstanding masticatory forces and should be always considered [25].

#### 7.1.2. Excessive Force during Root Canal Obturation

Excessive pressure during lateral or vertical compaction may result in a VRF [13,25,45,46,47]. The wedging forces may initiate stresses and strains, and further lead to root fracture [48,49,50,51]. However, other studies have demonstrated that the prevalence of VRFs caused by lateral condensation force is relatively low [48,52]. The maximum stress and strain produced during root canal obturation were investigated, and the results were significantly lower than those observed with condensation force, which could cause root fractures. Thus, condensation forces may not be the direct cause of root fracture. A weaker radicular structure tends to generate initial cracks, which could lead to root fracture even after the application of normal force [46].

#### 7.1.3. Excessive Post Space Preparation

Post space preparation may weaken the radicular structure and further result in VRFs [13,47,53,54,55]. Post space design should minimize the removal of the intact radicular dentin structure. Posts should be placed into the canal with minimal force [28]. Any intracanal wedging effects generated during treatment procedures should be avoided, because these may exceed the elasticity of dentin [25]. Fiber posts possess a similar modulus of elasticity to dentin. Studies have suggested that fiber posts could reduce root fractures and increase the survival rate of endodontically treated premolars [56,57].

### 7.2. Predisposing Factors

#### 7.2.1. Loss of Remaining or Internal Tooth Structure

Preservation of the remaining and internal tooth structure should be emphasized when restoring endodontically treated teeth [58,59]. Endodontically treated teeth are more susceptible to VRFs because they are usually associated with tooth or root structural loss.

#### 7.2.2. Specific Anatomies of the Susceptible Roots

Roots with a narrow mesiodistal width, such as upper premolars and mandibular molars, are more susceptible to VRFs [4,54,60,61,62]. A VRF usually initiates from the area of the canal wall with the greatest curvature as a result of asymmetrical stress distribution [63]. Irregularities in the inner canal surface may increase localized stress [63]. Canal shape, root shape and dentin thickness have been investigated to confirm which affects the tensile stress distribution the most. Among the three factors, canal shape was determined to be the most important. The conclusion of the research was that an ovoid root and ovoid canal, combined with reduced proximal dentin thickness, would increase the occurrence of VRFs [27].

#### 7.2.3. Age-Related Microstructural Changes

**Figure 4:**
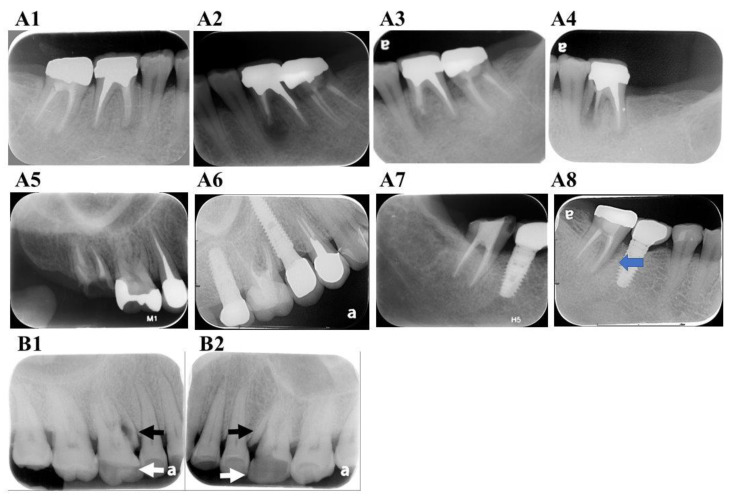
**Clinical cases of VRF.** (**A1**) A 31-year-old female patient showed 46 clinical symptoms even after endodontic retreatment and crown fabrication in January 2006. A VRF in the mesial root with deep pocket was noted after surgical exploration. (**A2**) In the same patient, a periapical radiograph of 36 was taken immediately after apical surgery of the mesial root in December 2011. Apical radiolucency was noted. (**A3**) Complete healing of apical lesion over the mesial root of 36 in May 2012. (**A4**) Sinus tract formation and a deep periodontal pocket with VRF of the mesial root of 36 was found in October 2013. (**A5**) Radiograph picture of 15, 16 and 17 in June 2016. Endodontic retreatment of 17 was completed. Endodontic treatment and crown procedure performed several years ago in the local dental clinic. (**A6**) Radiograph of the same region in November 2020; 15 and 17 were extracted due to VRFs and replaced with implants during this period. (**A7**) Endodontic treatment of 47 was completed in May 2013. (**A8**) VRF of 47 with deep pocket and radiolucency (blue arrow) around the whole mesial root of 47 in November 2020; (**B**) VRFs (black arrows) in non-endodontically treated teeth usually present along with an attrited occlusal surface (white arrows). (**B1**) 16 and (**B2**) 26 in the same patient.

Increases in brittleness and reductions in fracture resistance are expected with aging [64]. Thus, the teeth of older patients may be more susceptible to root fractures than those of younger patients [65]. Another study suggested that fractures are significantly associated with sclerotic dentine formation, which increases with aging. Sclerotic dentin displays lower toughness and reduced flexibility in older people. Thus, age-related microstructural changes may also be an underlying cause of VRFs in endodontically treated teeth [66]. However, some clinical patients showed multiple VRFs in different teeth during sequential follow-up periods (Figure 4A1–A8), suggesting the presence of genetic and developmental factors that may make the intrinsic dentin structures more susceptible to VRFs.

#### 7.2.4. Implant-Related VRFs

An implant-protective occlusion, which minimizes the occlusal loading on the implant, may make the adjacent natural teeth vulnerable to greater occlusal forces [67,68,69]. Endodontically treated teeth have been reported to exhibit lower fracture resistance than vital teeth [70]. Therefore, the possibility of implant-related VRFs in endodontically treated adjacent teeth has been suggested [71].

#### 7.2.5. Repetitive Heavy and Stressful Chewing Habits

Non-endodontically treated VRF teeth are usually related to occlusal factors. The Chinese population presents some unique chewing habits. For example, the chewing of betel quid, bones in meat, or food that is not easily sheared are risk factors that may predispose teeth to VRFs in non-endodontically treated teeth [2,3,4,19,22,72]. Chewing betel quid (a product of the areca nut, with coarse fibers) is more popular in males in Taiwan [73]. This oral habit is reported to contribute to VRFs in Chinese populations [3,74].

## 8. Clinical Features

The clinical features of VRFs are extremely variable. The symptoms and signs may be different depending on the extent of the fracture line, the time after fracture, the architecture of the surrounding apparatus and the inflammatory stage [32].

### 8.1. Pain

A history of discomfort or pain when biting is a common finding, and is accompanied by localized chronic inflammation. Dull pain or a mild degree of discomfort may arise, but severe pain is relatively rare [13,32].

### 8.2. Soft Tissue Swelling and Sinus Tract

The sinus tract of a VRF tooth may be coronally located closer to the gingival margin than the apical area. Sinus tracts may be situated some distance from the fractured tooth. Thus, the insertion of a gutta-percha point into the sinus tract to trace the offending tooth assists in diagnosis [32]. If the gutta-percha cone appears parallel to the periodontal ligament (PDL), a VRF is highly suspected. This unique tracing pattern provides an important diagnostic difference between a VRF and other endodontic or periodontal pathologies [33].

### 8.3. Deep Periodontal Probing Depth

A deep, narrow, isolated periodontal pocket close to the fracture site was discovered in 64–93% of VRF cases [13,14,25,30]. A periodontal pocket is generally formed as bony destruction is exacerbated during the progression of a VRF [25]. However, in its early stage, no osseous defect or deep probing depth may be evident.

### 8.4. Attrited Occlusal Surface

Most of the non-endodontically treated VRF teeth showed moderate to severe attritions in relatively intact crowns with minimal restorations [4,19]. The attrited occlusal surface may indicate excessive, repetitive and heavy masticatory stress, which may further lead to root fractures in these patients [4,19].

### 8.5. Other Clinical Symptoms and Signs

Pain in response to percussion, palpation and mastication may be reported by these patients [6,13,36]. The common clinical symptoms and signs of VRFs elucidated in previous studies are shown in Table 1. 

Radiographic examination is essential to the diagnosis of a VRF. Radiographic changes in the surrounding apparatus can sometimes be the only clue of a root fracture. An immediate radiographic diagnosis can be made if separated root fragments [13,76] or a hair-like radiolucency, interpreted as a crack in the dentin, are recognized [72].

Possible radiographic changes in VRFs include the following: displacement of a fractured fragment, a radiolucent line within the root canal, an unusual space between the canal wall and intracanal material, a widening PDL space, a periradicular radiolucent halo, angular bony destruction, a step-like bone defect, a V-shaped diffuse bone defect, root resorption that corresponds to the fracture line, widening of the root canal space, endodontic failure after healing has occurred, or no evident radiographic finding.

## 9. Radiographic Characteristics

### 9.1. Displacement of Fractured Fragment

**Figure 5:**
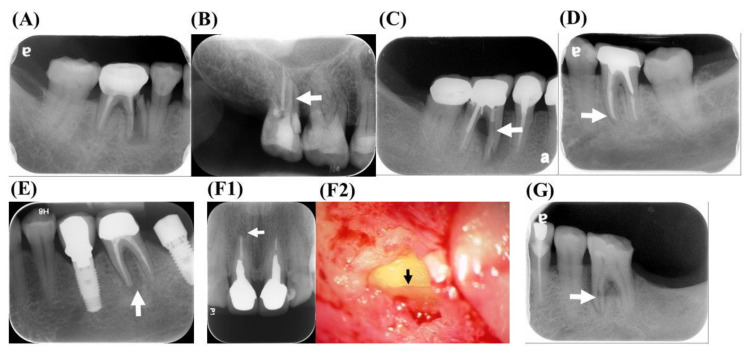
Radiographic features of the VRFs. (**A**) Displacement of fractured root fragment, (**B**) radiolucent lines within the root canal (white arrow), (**C**) unusual radiolucent space between the canal wall and intracanal material (white arrow), (**D**) widening of the periodontal ligament (PDL) space (white arrow), (**E**) periradicular radiolucent halo (white arrow), (**F1**) root resorptions corresponding to the fracture line (white arrow); (**F2**) VRF was further diagnosed via exploratory surgery (black arrow) and (**G**) a widening of the root canal space (white arrow).

When the root fragments are separated, a root fracture is visible on the radiographic image (Figure 5A) [25]. The proliferation of granulation tissue would cause the movement of the fragment away from the original tooth structure and is a definitive indicator of a root fracture [32].

### 9.2. Radiolucent Line within the Root Canal 

A root fracture may be displayed as an unusual and wide radiolucent line in the root canal space or the root filling material (Figure 5B). In endodontically treated teeth, the fracture line can sometimes be observed more clearly in the radiograph after the removal of the root canal filling material.

### 9.3. Unusual Space between the Canal Wall and Intracanal Material 

The mild displacement of VRF fragments could create a radiolucent space adjacent to the root filling material in a well-obturated canal (Figure 5C). Posts are usually tightly cemented to the canal wall. If a suspicious radiolucent space is present between the post and the root canal space, a VRF may have occurred [32].

### 9.4. Widening PDL Space 

An enlargement of the PDL around the root apex or even the whole root surface may indicate that the tooth is vertically fractured (Figure 5D) [1,4,13,32]. This radiographic description is quite different from that of typical endodontic lesions, which are limited to the apical area and do not include the destruction of the lamina dura along the periradicular surfaces.

### 9.5. Periradicular Radiolucent Halo

Radiolucent halos represent periradicular rarefaction, which can be observed on the lateral or even opposite side of the root surface (Figure 5E) [14,31,76,77]. Halo radiolucency is recognized as one of the most common radiographic characteristics of a VRF [1,25,28,31,78]. In addition, J-shaped lesions around the root have also been identified as a radiographic feature of a VRF [11,36].

### 9.6. Angular Bony Destruction

Angular periodontal defects may extend from the marginal bone to the fracture line in a VRF. This depends on the extent of the fracture and the inflammation [25]. Osseous defects break down faster in areas of thin buccal bone plate, such as around the maxillary premolars and the mesial roots of the mandibular molars [28,30,33].

### 9.7. Step-Like Bone Defect

Step-like bone destruction may develop if the vertical fracture line extends obliquely through the root or does not appear in the apical portion [31,32,79]. Shifting 15 degrees in the mesial or distal direction may assist in the observation of the defect. However, step-like bony destruction is not a definite indication of a VRF. Besides a VRF, canal perforations and endodontic lesions are also possible. Thus, the exact diagnosis of a VRF needs to be confirmed with other diagnostic methods [32].

### 9.8. V-Shaped Diffuse Bone Defect

V-shaped bone destruction may derive from the discrepancy between buccal and lingual bony destruction. This kind of destruction is wide at the crestal bone and narrows toward the root apex [31]. If diffuse bone loss occurs in a single root or tooth, a VRF is highly suspected [32].

### 9.9. Root Resorption Correspond to the Fracture Line (Figure 5F1,F2)

Root resorption forming a V-shaped notch at the root apex has been reported as a feature of a VRF. The root canal filling material may disintegrate when there are irregular resorptive defects in the root [80].

### 9.10. Widening of the Root Canal Space (Figure 5G)

The root canal usually becomes subtle as it extends to the apical region. Sudden changes in the radiodensity of the root canal, or the unusual widening of the canal space, may indicate a VRF, especially in non-endodontically treated teeth [22,26].

### 9.11. Endodontic Failure after Healing has Occurred

If an endodontically treated tooth deteriorates rapidly after many years without symptoms, or if rarefaction reoccurs without other specific problems, a VRF should be considered [32].

### 9.12. No Evident Radiographic Finding

About 13–14% of the VRFs show no detectable periapical or lateral radiolucency on the radiograph [25,30]. This may be because the bony destruction had not penetrated into the cortex yet, and the results were based on two-dimensional radiographs.

The radiographic bony defect patterns and features of VRFs are listed in Table 2. A comparison and summary of VRFETT and VRFNETT are given in Table 3.

## 10. Treatment and Prevention of VRFs

In addition to root amputation, hemisection and extraction [82,83], VRFs have been treated via various intraoral and extraoral methods [24,32,33,36,72,84,85]. According to the extent and location of the fracture, different treatment strategies have been reported to preserve the tooth. For example, the fusing of the interface by applying a CO_2_ laser [86], the use of a calcium hydroxide dressing to aid healing [87,88], removing the fracture fragment from single-rooted teeth [89,90,91], re-cementing with a glass–ionomer material [92,93], bonding with multiple adhesive resins [94,95,96,97,98,99,100,101,102,103], or sealing with bioceramic materials [104,105,106]. A case study of applying 4-methacryloxyethyl trimellitate anhydride/methyl methacrylate-tri-n-butyl borane resin to bond the fracture line via an intentional replantation was successful [103]. Some clinicians also employed guided tissue regeneration to improve the outcome [92,93,100,101,104,105]. The different treatment strategies and their outcome prognoses for VRFs are elucidated in Table 4, Table 5 and Table 6.

Some studies also provided clinical suggestions for preventing VRFs. The clinicians should use the dental instruments as conservative as possible in order to avoid root fracture [46,92]. Minimizing the forces applied during endodontic or prosthetic procedures was significant in reducing the possibility of VRF [27]. Minimal or conservative root canal enlargement and flare preparation had been suggested [59]. Intracoronal and intraradicular restorations should be placed passively with caution [13]. For patients with the habit of bruxism or clenching, night guards were able to provide some protection to minimize the risk of VRF [33].

## 11. Conclusions

VRFs in non-endodontically and endodontically treated teeth share some common factors, such as age-related microstructural changes, the specific anatomies of the susceptible roots, biting pain, deep periodontal pockets and periodontal or periradicular radiolucency. The attrition of the occlusal surface is a common feature in VRFs of non-endodontically treated teeth. The possible etiologies are related to iatrogenic problems or masticatory and occlusal factors. Radiographic assessment, CBCT imaging and visual inspection during exploratory surgery are used for diagnosis.

The value of this article is its provision of an overview of the current knowledge of VRFs in endodontically and non-endodontically treated teeth concomitantly. It provides an opportunity to improve the identification and treatment principles of VRFs. Further investigations regarding the mechanism of VRFs from basic and clinical aspects should be conducted. In addition to this, the long-term prognosis of various therapeutic schemes should be assessed to avoid inappropriate treatment and frustrated results.

## Figures and Tables

**Table 1:** Clinical symptoms and signs of a vertical root fracture (VRF).

Author	Number of Teeth	Periodontal Pocket	Pain	Swelling Abscess	Sinus Tract
Meister et al., 1980 [13]	32	93%	66%	28%	13%
Chan et al., 1998 [19]	64	84%	52%	30%	11%
Tamse et al., 1999 [30]	92	67%	55%	34%	35%
Cohen et al., 2006 [6]	227	40%	Pain on percussion: 69% Pain on palpation: 69% Pain on mastication: 61%	15%	18%
PradeepKumar et al., 2016 [1]	197	81%	Pain on percussion: 60% Pain on palpation: 62%	67%	
Liao et al., 2017 [22]	65	91%	NA	NA	NA
Walton et al., 2017 [36]	42	66%	No to mild pain: 100%	77%	31%
Von Arx and Bosshradt, 2017 [75]	30	40%	Pain: 60% Percussion sensitivity: 6% Palpation sensitivity: 6%	23%	46%
See et al., 2019 [11]	61	57%	Tenderness to percussion: 27% Tenderness to palpation: 29%	36%	60%

NA: no data available.

**Table 2:** Radiographic features of a VRF.

Author	Number of Teeth	Halo Radiolucency	Lateral Radiolucency	Apical Radiolucency	Fractured Root Displacement	Angular Defect	Normal Appearance	Other Findings
Meister et al., 1980 [13]	32	75%		22%	3%	NA	NA	
Nicopoulou- Karayianni et al., 1997 [77]	22	45%	27%	5%	NA	0%	5%	
Chan et al., 1998 [19]	64	NA	NA	27%	20%	63%	NA	PDL widening: 39% Root canal space widening: 25%
Tamse et al., 1999 [30]	51	57%	14%	4%	NA	14%	2%	
Tamse et al., 1999 [76]	92	39%	24%	24%	NA	NA	13%	
Tamse et al., 2006 [78]	49	37%	29%	10%	NA	6%	8%	
Cohen et al., 2006 [6]	227	50%		21%	27%	NA	NA	
Liao et al., 2017 [22]	65	NA	NA	80%	43%	95%	NA	
Walton et al., 2017 [36]	42	NA	NA	21%	17%	11%	21%	
Von Arx and Bosshradt, 2017 [75]	30	36%		53%	NA	NA	10%	
See et al., 2019 [11]	61	50%	14%	26%	NA	NA	4%	

NA: no data available.

**Table 3:** Comparison and summary of VRFs in endodontically treated teeth (VRFETT) and VRFs in non-endodontically treated teeth (VRFNETT).

Category	VRFETT	VRFNETT
Prevalence	2–25% [7,8,9,10,11,13,14,15,16,17]	Not reported
Gender	No preference in gender [1,4,6,18]	Male [2,4,19,20]
Age	Predominantly > 40 years old [3,4,6,14,19] Mean age: Non-endodontically treated group>Endodontically treated group [2,3,19,20,22]	
Tooth distribution	Maxillary premolars and mandibular molars [23,24,25]	Maxillary and mandibular first molars in the Chinese population [2,4,19,20,26]
Root distribution	Premolars and mesial roots of mandibular molars [14,23,24,25]	Mesiobuccal roots of maxillary molars and mesial roots of mandibular molars [2,3,4,19,20,26]
Etiology and predisposing factors	**Iatrogenic factors ** Excessive tooth structure removal or over-preparation during instrumentation [28,33] Excessive forces during obturation [13,25,46,47] Excessive post space preparation [13,47,53,54,55] **Predisposing factors** Loss of remaining or internal tooth structure [58,59] Implant-related VRF [71]	Repetitive heavy and stressful chewing habits [2,3,4,19,22,72]
	Specific anatomies of the susceptible roots [4,54,60,61,62]	
	Age-related microstructural changes [65,66,81]	
Clinical features	Mostly in endodontically treated teeth [4,14] Dull pain or mild discomfort [13,32] Soft tissue swelling [32,33] Coronally positioned or multiple sinus tracts [14,28,30,31,32] Biting pain [34]	
		Attrited occlusal surface [4,19]
	No pain or notable changes [6,13,36] Deep periodontal pocket [13,14,25,30]	
Radiographic characteristics	Displacement of fractured fragment [25,32]	
	Radiolucent longitudinal lines within the root adjacent to the canal [31,81] Widening of PDL space [1,4,13,22,32] Periradicular radiolucent halos or angular bony destruction [1,14,25,31,76,77,78]	
	Unusual space between the canal wall and intracanal material [32] Step-like bone defects [31,32,79] V-shaped diffuse bone defects [32] Root resorptions correspond to the fracture line [80] Endodontic failure after healing has occurred [32]	Widening of the root canal space [22,26]

**Table 4:** Research regarding the application of CO_2_ laser, intracanal medication or removing the fracture fragment in treating VRFs.

Author	Number of Teeth	Status of the VRF Teeth	Method	Management or Material Used to Seal the Fracture Interface	Follow-Up	Prognosis
Sinai et al., 1978 [89]	1	VRFETT	Intraoral	The root segment, canal filling material and the granulomatous tissue were all removed.	10 years	Bone formation was observed at 7 months follow-up. However, the long-term outcome was unfavorable.
Vertucci, 1985 [90]	1	VRFETT	Intraoral	Removal of a major portion of the buccal half of the root and applying 20% citric acid solution for 5 min on all exposed root surfaces.	3 years	The tooth functioned normally without periodontal defect and radiographic pathosis. However, the author considered that the long-term prognosis remained questionable.
Stewart, 1988 [87]	3	1 VRFETT 2 VRFNETT	Intraoral	Canal dressing with calcium hydroxide plus the contrast medium. At least 9 to 12 months were needed to present bone formation and more cementum for healing. Then, the root canal was obturated with gutta-percha.	4 months to 10 years	Healing of the periradicular tissue and increasing bony density were noted.
Matusow, 1988 [91]	1	VRFETT	Intraoral	Strip the fused fractured mesial root and leave the distal root fragment in the molar of a bridge abutment.	14 months	The tooth was asymptomatic and showed bone repair.
Barkhordar, 1991 [88]	1	VRFNETT	Intraoral	Use calcium hydroxide dressing to induce healing of fractured roots. Glass–ionomer cement was further used as a root canal sealer to bond the fracture fragment.	6 months	Healing of the osseous defect was observed.
Dederich, 1999 [86]	1	VRFETT	Intraoral	Apply CO_2_ laser fusion of the fracture interface and place a compressed collagen matrix barrier.	1 year	No inflammation, pocket reduction and increased radiodensity at the osseous defect.

**Table 5:** Research regarding the re-cementation of VRFs with multiple adhesive resins.

Author	Number of Teeth	Status of the VRF Teeth	Method	Management or Material used to Seal the Fracture Interface	FOLLOW-UP	Prognosis
Oliet, 1984 [94]	3	1 VRFNETT 2 VRFETT	Extraoral and intentional replantation	Re-cementation of the fracture fragment with cyanoacrylate.	3 to 16 months	Although the teeth functioned normally, the long-term prognosis remained poor.
Funato et al., 1999 [95]	1	VRFETT	Intraoral	4-META/MMA-TBB dentin-bonded resin	6 months	The tooth was asymptomatic and showed reduced radiolucent area.
Sugaya et al., 2001 [96]	23	VRFETT	Group A: Intraoral Group B: Extraoral and intentional replantation	4-META/MMA-TBB dentin-bonded resin	22 to 33 months	Group A: 9 out of 11 cases with good prognosis Group B: 9 out of 12 cases with good prognosis
Hayashi et al., 2002 [97]	20	VRFETT	Extraoral and intentional replantation	4-META/MMA-TBB dentin-bonded resin	4 to 45 months	Survival rates were 83.3% at 12 months and 36.3% at 24 months.
Kawai et al., 2002 [98]	2	VRFETT	Extraoral and intentional replantation	Apply adhesive resin cement to bond the fracture interface.	3 years	The teeth were asymptomatic and displayed bone regeneration.
Hayashi et al., 2004 [99]	26	VRFETT	Extraoral and intentional replantation	4-META/MMA-TBB dentin-bonded resin	4 to 76 months	Survival rates were 88.5% at 12 months, 69.2% at 36 months and 59.3% at 60 months.
Öztürk and Ünal, 2008 [100]	1	VRFETT	Extraoral and intentional replantation	Apply self-etching dual-cured adhesive resin cement and place a membrane.	4 years	The tooth was asymptomatic and bone regeneration was observed.
Özer et al., 2011 [45]	3	VRFETT	Extraoral and intentional replantation	Self-etching dual-cure adhesive resin cement	2 years	The teeth were asymptomatic and showed reduced periapical radiolucency.
Nogueira Leal da Silva et al., 2012 [101]	1	VRFETT	Intraoral	Bond with composed resin and place a synthetic hydroxyapatite graft.	2 years	The tooth showed no symptom and sign.
Moradi Majd et al., 2012 [102]	1	VRFETT	Extraoral and intentional replantation	Prepare the fracture line with an ultrasonic device and seal with dual-curing resin.	12 months	The tooth was asymptomatic, and the apical radiolucency reduced in size.
Okaguchi et al., 2019 [103]	6	VRFETT	Extraoral and intentional replantation	4-META/MMA-TBB dentin-bonded resin	33 to 74 months	Tooth function was normal with successful clinical outcome and healing of radiolucent lesions.

4-META/MMA-TBB: 4-methacryloxyethyl trimellitate anhydride/methyl methacrylate-tri-n-butyl borane.

**Table 6:** Research regarding the re-cementation of VRFs with glass-ionomer materials or sealing with bioceramic materials.

Author	Number of Teeth	Status of the VRF Teeth	Method	Management or Material Used to Seal the Fracture Interface	Follow-Up	Prognosis
Trope et al., 1992 [92]	1	VRFETT	Extraoral and intentional replantation	Biocompatible glass–ionomer bone cement in conjunction with an expanded polytetrafluoroethylene (Gore-Tex) membrane.	1 year	The tooth functioned normally without periodontal pocket and exhibited good healing outcome.
Selden, 1996 [93]	6	VRFETT	Intraoral	Apply silver glass–ionomer cement to bond the fracture fragment and perform guided tissue regeneration.	2 to 12 months	Five cases failed within 2 to 11 months. The other one was asymptomatic but failed at 1 year due to exacerbation of the fracture line.
Floratos and Kratchman, 2012 [105]	4	VRFETT	Intraoral	The fracture line was removed by resecting the root fragment. Retrograde preparation and retrograde filling were performed with MTA. An absorbable collagen membrane was covered over the bone defect.	8 to 24 months	The teeth were asymptomatic. Periapical healing with periodontal ligament re-formation was noted.
Hadrossek and Dammaschke, 2014 [106]	1	VRFETT	Extraoral and intentional replantation	Prepare the fracture gap with a small diamond bur and fill with Biodentine. Then, replant the tooth with fixation.	24 months	The tooth was asymptomatic, and the periodontal pocket returned to normal.

MTA: mineral trioxide aggregate.

## Data Availability

All data in this paper are available in the cited references.
